# Consumers’ Quality Perception and Acceptance of Suboptimal Food: An Online Survey in Selangor and Kuala Lumpur, Malaysia

**DOI:** 10.3390/foods12152824

**Published:** 2023-07-25

**Authors:** See Meng Lim, Hanbin Law, Siew Siew Lee

**Affiliations:** 1 Nutritional Sciences Programme & Centre for Community Health Studies (ReaCH), Faculty of Health Sciences, Universiti Kebangsaan Malaysia, Kuala Lumpur 50300, Malaysia; 2 School of Biosciences, Faculty of Science and Engineering, University of Nottingham Malaysia, Semenyih 43500, Malaysia

**Keywords:** food waste reduction, suboptimal food, perception, acceptance, consumer behaviour, sustainable consumption

## Abstract

Suboptimal food is defined as physically imperfect food that deviates from the norm in terms of appearance without compromising its intrinsic quality or safety. Consumers’ quality perception and acceptance of suboptimal food contribute to food waste. Therefore, this study aims to explore consumers’ quality perception and acceptance of suboptimal food and the factors associated with the acceptance of suboptimal food. An online survey was conducted among 414 consumers residing in Selangor and Kuala Lumpur, Malaysia, through convenience sampling. They completed an online questionnaire asking for sociodemographic information, quality perception and acceptance of suboptimal food, and information related to food waste. Only 11.4% of consumers chose suboptimal foods, with visually deviated suboptimal foods (apples with brown spots) having the lowest acceptance (9.9%). Consumers perceived suboptimal foods as unattractive and that they should be consumed quickly. Malays were less likely to accept suboptimal foods, while middle-income households were more likely to accept suboptimal foods at home. In conclusion, consumers have a low acceptance of suboptimal food, and suboptimal food was perceived as unappealing and that it should be consumed quickly. Notwithstanding the findings that emerge from this, the results may lack generalisability to the wider population as only a convenience sample was used.

## 1. Introduction

Food waste is a major problem worldwide, contributing to both global warming and world hunger. Each year, nearly 1.3 billion tonnes, or about one-third of all food produced, is wasted globally [1]. Composting food waste releases harmful greenhouse gases that account for 3.3 billion tonnes per year, or 7% of the total greenhouse gas emissions into the atmosphere [2]. However, hunger still affected 828 million people worldwide in 2021 [3]. In Malaysia, according to the Solid Waste Management and Public Cleansing Corporation (SWCorp), 17,000 tonnes of food waste are produced every day, of which 4050 tonnes (24%) are still edible and avoidable. The total food waste in Malaysia can feed 3 million people three times a day [4]. This discrepancy between food waste and hunger is unacceptable and therefore requires immediate action.

Suboptimal food is defined as physically imperfect food that deviates from the norm in terms of appearance without compromising its intrinsic quality or safety. The deviation can be visual (e.g., imperfect appearance), temporal (e.g., approaching expiry date), or peripheral (e.g., packaging damage) [5]. The rejection of suboptimal food by consumers contributes to food waste, with consumers playing a particularly large role in causing food waste by rejecting suboptimal food [6]. Consumers often expect visually perfect food products, immaculate packaging, and fresh food products. As a result, food that deviates even slightly from these perceived ideals tends to be rejected and discarded, contributing significantly to unnecessary food waste. Several previous studies have shown that the consumer acceptance of suboptimal foods is low because consumers perceive the quality of suboptimal foods to be low [7,8,9]. For example, visually deviant suboptimal foods are perceived by consumers as unattractive, perishable, and poor in taste [10]. Food that is close to its expiry date is considered not fresh [7]. Consumers also tend to avoid purchasing products with defective packaging because they fear the risk of contamination and the resulting risks to safety and health [11].

Sociodemographic factors can influence consumer behaviour and acceptance of suboptimal foods [7,8,10]. Various sociodemographic characteristics, including age, gender, education, and ethnicity, are associated with the acceptance of suboptimal foods [12]. For example, studies have shown that consumers with lower levels of education have a lower acceptance of suboptimal foods than consumers with higher levels of education [7,8,13]. The acceptance of suboptimal food also differs according to the phase of the decision (at purchase or consumption). A previous study found that consumers are generally more receptive to suboptimal foods at home (during the consumption phase) than in supermarkets (during the purchasing phase) [8]. In addition, the awareness of food waste may also influence the acceptance of suboptimal foods. A previous study reported that consumers who consider the problem of food waste to be more important have a higher acceptance of suboptimal food [8].

Numerous studies have been conducted on the consumer acceptance of suboptimal foods in various countries, including Denmark, Germany, Norway, Sweden, the Netherlands [8,14], Sri Lanka [15], Brazil [9], and Uruguay [7]. In Malaysia, however, existing studies mainly focus only on the problem of food waste alone [16,17], while suboptimal foods, which are also major contributors to food waste, have hardly been studied. To the best of our knowledge, there is only one published study on suboptimal food in Malaysia by Abd Razak et al., (2022). The study, which involved 399 consumers living in Selangor, Malaysia, found that consumers’ purchase decisions for suboptimal foods were related to the acceptability of suboptimal foods and consumers’ perceptions of the perceived quality and safety of suboptimal foods [18]. Therefore, more studies should be conducted in Malaysia to gain a better understanding of consumers’ perceptions towards suboptimal foods so that food waste due to the rejection of suboptimal food can be reduced.

### Research Hypothesis and Framework

Consumers’ perception of the quality of suboptimal foods plays an important role in influencing the acceptance of these foods. Consumers’ perception of quality is shaped by various factors, including sensory attributes, visual appearance, taste, texture, freshness, and safety. If consumers perceive suboptimal food as inferior compared to optimal alternatives, this may negatively impact their willingness to accept them. However, it is currently unclear how Malaysian consumers perceive the quality of suboptimal foods that deviate visually, temporally, or peripherally. Therefore, we predicted the following:

**Hypothesis 1 (H1).**  *Malaysian consumers have a low perception of quality for all three forms of suboptimal food*.

**Hypothesis 2 (H2).**  *Malaysian consumers have a low acceptance of all three forms of suboptimal food*.

Sociodemographic factors are one of the prominent factors affecting consumers’ acceptance of suboptimal food. Factors, including gender, monthly household income, household size, and education level, have been shown to influence the acceptance of suboptimal food. However, it is currently unclear whether Malaysian consumers with different sociodemographic characteristics accept suboptimal foods that deviate in terms of their visual, temporal, and peripheral differences. In addition, consumers’ acceptance of suboptimal food may differ at different phases of the decision: at the time of purchase (in the supermarket) or at the time of consumption (at home). Therefore, we predicted the following:

**Hypothesis 3 (H3).**  *Acceptance of suboptimal food differs by sociodemographic characteristics*.

**Hypothesis 4 (H4).**  *Acceptance of suboptimal food at home and in the supermarket differ by sociodemographic characteristics*.

**Figure 1:**
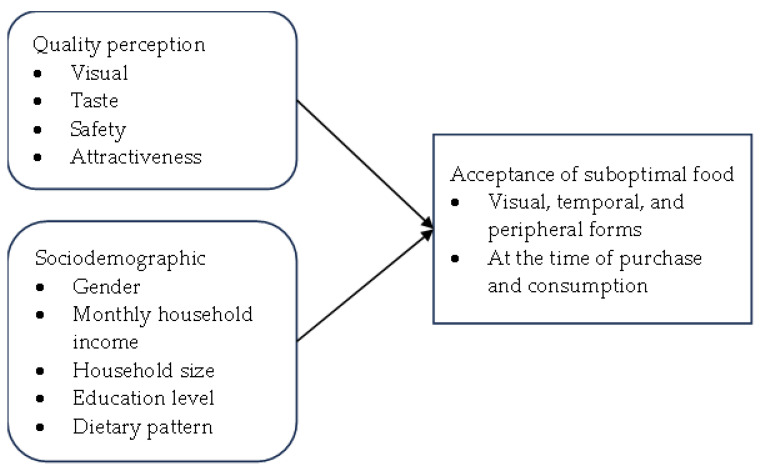
Proposed research framework.

A research framework based on these hypotheses was proposed in this study (Figure 1). This study has two main objectives. First, the study aimed to examine consumers’ perceptions of quality and the acceptance of suboptimal food products that deviate in visual, temporal, and peripheral aspects. Secondly, this study compared the acceptance of suboptimal foods at home or in the supermarket with the sociodemographic characteristics of consumers. To achieve these objectives, an online survey was conducted with a total of 414 Malaysian adults aged 18 to 59 years old living in Selangor or Kuala Lumpur. Online questionnaires were used to collect information on sociodemographic and quality perceptions and the acceptance of suboptimal food. Three different forms of suboptimal foods were assessed in this study, including visual (i.e., apples with brown spots), temporal (i.e., milk approaching or past its “best-before” date), and peripheral (i.e., biscuits with packaging damage and partially broken). The findings of this study add new insights to the existing body of knowledge on suboptimal food in the Malaysian context.

## 2. Materials and Methods

### 2.1. Study Design

An online survey was conducted using Google Forms and distributed across various social media platforms, including WhatsApp, Facebook, Instagram, and Telegram. Consumers were asked to contribute to the research by completing the questionnaire and sharing it with their social contacts. The data were collected from September to November 2022. Malaysians aged 18 to 59 years old living in Selangor or Kuala Lumpur with experience in buying food from grocery shops or supermarkets were invited to participate in the study. Selangor and the Federal Territory of Kuala Lumpur were selected as study sites due to their high population density and rapid urban development, which could contribute to higher food wastage [19]. Ethical approval to conduct this study was obtained from the Research Ethics Committee of the Universiti Kebangsaan Malaysia (JEPUKM-2022-505). Informed consent was obtained online before consumers answered the questionnaire.

### 2.2. Questionnaire

Sociodemographic information, including gender, age, date of birth, ethnicity, state, marital status, monthly household income, household size, highest level of education, and dietary habits, were collected. The quality perception and acceptance of suboptimal food, awareness of food waste, estimation of food waste in the household, and perceived importance of reducing food waste were assessed using a questionnaire adapted from Aschemann-Witzel et al., 2021 [14]. The questionnaire was available in the English language, which was then translated into Malay language using forward and backward translation methods. Prior to data collection, the questionnaire was checked twice for clarity and comprehensibility, and a content validity test was conducted. The scale-level content validity index (S-CVI) was 1.0 based on the average method and universal agreement method. 

For the quality perception and acceptance of suboptimal food, three suboptimal foods were examined, namely, apples with brown spots (visual), milk approaching or past its “best-before” date (temporal) (yesterday—at home, today—at the supermarket), and biscuits showing packaging damage and are partially broken (peripheral). Consumers were asked about their perceptions of the quality and acceptability of these three suboptimal foods in two situations: at home and in the supermarket. For the home situation, consumers were asked to imagine that they were choosing an apple at home from three options, namely “A” (optimal), “B” (suboptimal), or “none of these”. Then, consumers were asked how likely it was that Apple B (suboptimal) would be discarded in the garbage, on a scale of 0 to 10, where 0 indicated that the item would definitely be consumed, 5 an equal probability of consumption or disposal, and 10 that the item would definitely be disposed of. Next, consumers had to choose the description associated with Apple B (suboptimal) at home from a list of 12 check-all-that-apply (CATA) options. The options included good taste, bad taste, same taste as the other product, safe to eat/drink, unsafe to eat/drink, not attractive/enticing to eat/drink, suitable for adults, suitable for children, suitable for serving to guests, throw away, eat as soon as possible, and suitable for cooking. For the supermarket situation, consumers were asked to imagine that they were in a supermarket choosing an apple from three options, namely “A” (optimal), “B” (suboptimal), or “none of these” if the price was the same between “A” (optimal) and “B” (suboptimal). The sales promotion threshold was then determined, which refers to the minimum value of price promotion required to change consumers’ intention to purchase suboptimal food. Consumers were asked to indicate on a scale of 0 to 10 the maximum discount at which they would purchase Apple B (suboptimal), where 0 represented normal price or 0% discount, 5 represented half price or 50% discount, and 10 represented free or 100% discount. Next, consumers had to choose the description associated with Apple B (suboptimal) in the supermarket by using the 12 CATA options listed above. The same questions were repeated for milk and biscuits as the food items in home and supermarket situations (Supplementary Material).

Next, consumers were asked about their awareness of food waste, estimation of food waste in the household, and perceived importance of reducing food waste. The questions were: “What do you estimate to be the percentage of food waste in the global food supply chain?” and “What do you estimate to be the percentage of food waste in households compared to the amount of food purchased?”. Consumers were also asked to indicate the percentage of food that is thrown away in their households for five food categories: (1) fresh fruits and vegetables, (2) milk and dairy products, (3) bread and other bakery products, (4) meat and fish, and (5) ready meals/meals (leftovers). For the questions on the perceived importance of reducing food waste, consumers were asked to compare it to three other societal issues: (a) reducing obesity, (b) reducing environmental pollution, and (c) stabilising the economy, on a scale of 1 to 7, where 1 was “much less important” and 7 was “much more important”.

### 2.3. Data Analysis

The data were analysed using IBM SPSS Statistics for Windows (version 26.0, IBM Corp., Armonk, NY, USA). Descriptive statistics, including the mean, standard deviation, and percentage, were presented. The Likert scale was treated as interval data in this study. There were 50 respondents (12.1%) who selected neither “A” (optimal) nor “B” (suboptimal) in their food choices and were excluded from the analysis, except for sociodemographic characteristics. Correspondence analysis was performed to identify the quality perceptions for suboptimal foods. Differences between suboptimal food acceptance by location of food and sociodemographic characteristics were determined using Chi-square or Fisher’s Exact test. The Mann–Whitney U or the Kruskal–Wallis test was used to compare different types of suboptimal food and sociodemographic characteristics, as well as sociodemographic characteristics and the potential of suboptimal food being discarded and the promotion threshold to purchase suboptimal food. The association between quality perception and suboptimal food acceptance was determined with a binary logistic regression test. The statistical significance was set at *p* < 0.05.

## 3. Results

### 3.1. Sociodemographic Characteristics

A total of 414 consumers participated in this study. Table 1 shows the sociodemographic characteristics of the consumers. Most of the consumers were female (78.7%), aged between 18 and 29 years (94.4%), of Chinese ethnicity (64.3%), from Selangor (72.2%), single (95.4%), had a monthly household income of less than RM 4849 (≈1092 USD; 68.6%), lived with 4 to 6 people in the household (64.5%), had an education level of Matriculation/Diploma/STPM/Foundation (48.6%), and practised a diet that included meat and/or fish (74.6%).

### 3.2. Quality Perception and Acceptance of Suboptimal Foods

Table 2 shows consumers’ choices for three types of suboptimal food (A: optimal; B: suboptimal), the possibility of suboptimal food being discarded, and the promotion threshold to purchase suboptimal food. Overall, 11.4% of consumers chose suboptimal food, with most of them choosing suboptimal biscuits (14.4%), followed by suboptimal milk (10.0%) and suboptimal apples (9.9%). When it came to choices made at home, the percentage of consumers who chose suboptimal food was much higher (20.4%), with most of them choosing suboptimal biscuits (27.2%), followed by suboptimal apples (17.9%) and suboptimal milk (16.2%). In comparison, the percentage of consumers who chose suboptimal food at the supermarket was much lower (2.5%), with the total number of consumers choosing suboptimal apples, milk, and biscuits being 1.9%, 3.8%, and 1.6%, respectively. In addition, the suboptimal biscuits (3.66 ± 3.27) had the lowest probability of being discarded compared to suboptimal apples (4.56 ± 2.96) and suboptimal milk (7.20 ± 3.21). Furthermore, the promotion threshold for suboptimal biscuits (61.0 ± 27.9%) was the lowest, followed by suboptimal apples (66.7 ± 25.1%) and suboptimal milk (77.3 ± 25.5%).

**Figure 2:**
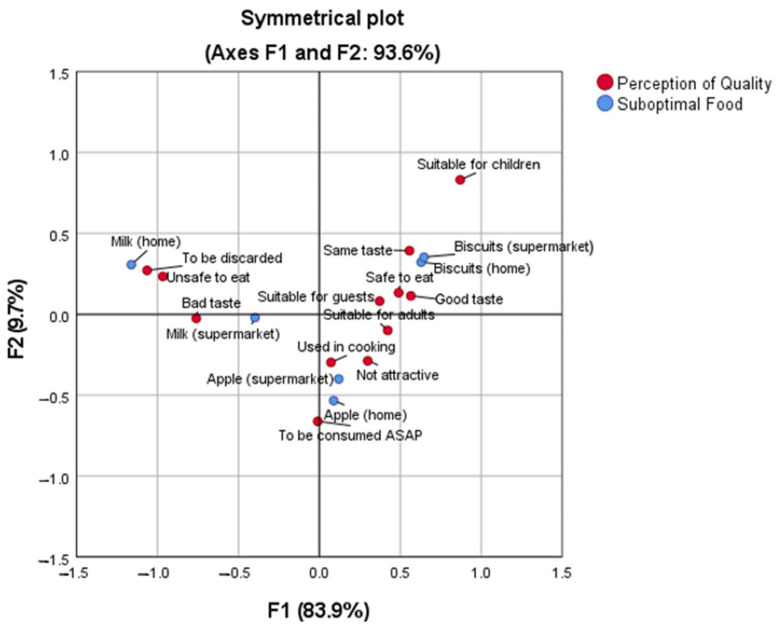
Correspondent analysis for the association between three types of suboptimal food at home or in the supermarket and their quality perception by consumers.

Figure 2 shows the descriptions associated with all three suboptimal foods in both home and supermarket settings. The results showed that suboptimal apples (visual) were perceived as unappetizing and should be consumed quickly. They were also seen as suitable for cooking and consumption by adults. Suboptimal milk (temporal) was associated with being discarded, being unsafe to drink, and having an unpleasant taste. In contrast, suboptimal biscuits (peripheral) were perceived as having a similar taste to non-suboptimal biscuits, tasting good, being safe to eat, suitable for guests, and suitable for adults and children. Overall, the quality perceptions were the lowest for suboptimal foods with time deviation (suboptimal milk) compared to visual (suboptimal apple) and peripheral (suboptimal biscuits) deviations.

### 3.3. Food Waste Awareness, Estimation of Food Waste in the Household, and Perceived Importance of Reducing Food Waste

Consumers estimated global food waste at 53.23 ± 21.05% and consumer food waste at 41.49 ± 21.57% (Table 3). The estimated household food waste was highest for prepared dishes/meals (26.42 ± 24.46%), followed by fresh fruits and vegetables (21.02 ± 20.57%), bread and other bakery products (16.46 ± 20.24%), milk and dairy (16.00 ± 19.43%), and meat and fish (11.54 ± 17.27%). The average estimate of food waste in consumers’ own households was 18.29 ± 17.00% for all five food categories. In addition, consumers rated the importance of reducing food waste the lowest compared to stabilising the economy (5.45 ± 1.48), reducing pollution (5.50 ± 1.43), and obesity (5.78 ± 1.25).

### 3.4. Comparision between the Acceptance of Suboptimal Food and Sociodemographic Characteristics

Table 4 shows the comparison between the acceptance of suboptimal food and the sociodemographic characteristics of consumers. The results indicated that there was a difference between ethnicity and acceptance of suboptimal foods overall or at home (*p* < 0.001), but the effect size was weak, with a value of phi ϕ = 0.082 (overall) or 0.128 (at home). Malays (7.1% overall, 11.9% at home) were less likely to accept suboptimal food compared to non-Malays (13.0% overall, 23.6% at home). In addition, monthly household income also showed a significant difference in the acceptance of suboptimal food at home (*p* < 0.05), but the effect size was weak, with a value of Cramér’s V ϕ-*c*. = 0.084. The middle-income group (RM4850–RM10959; 27.0%) was more likely to choose suboptimal foods at home compared to the lowest-income group (less than RM4849; 18.5%) and the highest-income group (more than RM10959; 20.5%).

### 3.5. Comparision between Sociodemographic Characteristics and the Possibility of Suboptimal Food Being Discarded and the Pomotion Threshold for Purchasing Suboptimal Food

Table 5 shows the comparison between sociodemographic characteristics and the possibility of suboptimal food being discarded and the promotion threshold for purchasing suboptimal food. The results showed that women (69.7%) required a higher promotion threshold than men (63.5%) for purchasing suboptimal food (*p* < 0.05). In addition, vegans or vegetarians had a higher possibility level of discarding suboptimal food (6.38) compared to flexitarians (5.85) or those who practised a varied diet (4.84), *p* < 0.001.

### 3.6. Association between Quality Perception and the Acceptance of Suboptimal Foods

Table 6 shows the association between quality perception and the acceptance of suboptimal food. For suboptimal apples, the quality perceptions associated with acceptance were ‘not attractive/tempting to eat’, ‘suitable for adults’, and ‘to eat as soon as possible’. For suboptimal milk, the quality perceptions associated with acceptance were “not attractive/tempting to drink”, “to be discarded”, and “consume as soon as possible”. As for suboptimal biscuits, the quality perceptions associated with acceptance were “same taste as the other product”, “not attractive/tempting to eat”, and “consume as soon as possible”.

## 4. Discussion

This study demonstrated that the consumer acceptance of suboptimal foods was low, with the lowest acceptance for visually deviated suboptimal food (apples), followed by temporally deviated suboptimal food (milk) and peripherally deviated suboptimal food (biscuits). However, the acceptance of suboptimal foods was significantly higher at home than in the supermarket. Suboptimal apples and suboptimal milk were associated with lower perceptions of quality, but the quality of suboptimal biscuits was perceived to be the same as that of optimal biscuits. We found that Malays were less willing to accept suboptimal food, while middle-income household consumers were more willing to accept suboptimal food at home. In addition, consumers on vegan or vegetarian diets were significantly more likely to discard suboptimal foods than consumers on flexitarian or varied diets.

Overall, the lowest consumer acceptance of suboptimal food in this study was for suboptimal apples, followed by suboptimal milk and suboptimal biscuits. The findings of de Hooge et al., 2017 showed that the acceptance of suboptimal apples was 23.6%, followed by suboptimal biscuits (38.3%) and suboptimal milk (48.7%) [8]. However, in this previous study, suboptimal milk was the most accepted suboptimal food [8], which contradicted the results of this study where suboptimal biscuits were the most accepted suboptimal food. Similarly, a study conducted by Yaqub (2016) showed that more consumers chose suboptimal milk over optimal milk, suggesting a higher acceptability of suboptimal milk [20]. While the exact reason for the difference in acceptability of different types of suboptimal food is unclear, the lower acceptability of suboptimal milk that deviates in time in this study could be due to the inability of some Malaysians to distinguish between the “best before” date and the “use by” date, as reported in a previous study in Selangor, Malaysia [21]. Consequently, consumers may choose to avoid suboptimal milk that is approaching its “best-before” date, despite the fact that the “best-before” date only indicates the quality of the food, not its safety [22]. This result may highlight the need to educate Malaysian consumers on how to read food labels.

In addition, we found that the suboptimal milk is more likely to be discarded and has the highest promotion threshold. The findings of Cao and Miao (2021) showed that suboptimal foods that deviate from the temporal dimension also have the highest probability of being discarded [5]. The studies by Chooi et al., 2022 and Fegola and Ismail (2018) showed that the most common reason behind the creation of food waste in Malaysia is disposing of food that has passed its expiry date is, which likely explains why suboptimal milk has the highest probability of being discarded [23,24]. However, the findings of de Hooge et al., 2017 showed that suboptimal apples have a higher likelihood of being discarded and the highest promotion threshold compared to suboptimal milk and biscuits [8]. This is because consumers have a negative quality perception of visually unattractive suboptimal apples compared to other types of suboptimal food, as reported in the study [8].

The results of this study on the acceptance of suboptimal foods at different locations were in line with previous studies which showed that the acceptance of suboptimal foods was greater at home than in the supermarket [8,14,25]. These results suggest that the acceptance of suboptimal food was lower at the point of purchase than at the point of consumption. The reason for the low acceptance of suboptimal foods at the point of purchase could be that consumers do not receive a discount for suboptimal food in the supermarket, so they are more likely to choose products with the best value for their money [26]. In contrast, if they have suboptimal food at home, they are less likely to waste suboptimal food because they have already bought it, and wasting food also means wasting their money [26].

In this study, the quality perception of suboptimal foods was consistent with the findings from previous studies. A study by Cao and Miao (2021) reported similar findings where suboptimal foods that have deviated in time were viewed as having the lowest quality perception and were associated with the highest health risks [5]. However, a study by Yaqub (2016) found that most consumers from Norway perceived suboptimal milk as tasty, safe to drink, and suitable for adults, children, and guests [20]. This could be because even if the food has passed its “best-before” date, it may still be edible or usable and not necessarily worth wasting [20]. On the other hand, the findings of Aschemann-Witzel et al., 2018 in Uruguay showed that suboptimal foods that deviated from the peripheral dimension were perceived as having the lowest quality compared to visually and temporally deviated suboptimal foods [7]. This could be due to the fact that Aschemann-Witzel et al., 2018 examined suboptimal foods that deviated from the peripheral dimension, such as dented canned peas [7], that were different from the suboptimal biscuits in this study, thus potentially affecting the outcome of the perception of suboptimal quality. In addition, the cultural differences and different economic statuses across countries could also influence consumers’ perceptions of suboptimal food, thus explaining the differences in results across individual studies [27,28].

Furthermore, the results of the comparison between the acceptance of suboptimal food and sociodemographic characteristics showed that middle-income households are most likely to choose suboptimal food at home. These results were not consistent with previous studies which showed that lower-income consumers are more likely to choose suboptimal foods [10,13]. This could be due to the fact that low-income groups have a higher neophobia scale for food [29,30], thus making them less acceptable to suboptimal foods since they are less familiar with them. On the other hand, the high-income group is more financially capable of buying perfect and optimal foods, so they also tend not to choose suboptimal foods [6]. Interestingly, we found that Malay consumers have a lower acceptance of suboptimal foods compared to other ethnic groups. Further studies are needed to explore the reasons for this finding.

In addition, the findings of this study on the promotion threshold for consumers purchasing suboptimal food by sociodemographic characteristics were consistent with the study by De Hooge et al., 2017 which found that women require a higher promotion threshold before buying a suboptimal food than men [8]. This may be because men do not care much about the price since they pay more attention to convenience and pay less attention when making purchasing decisions [31]. However, the results of this study on the probability of discarding suboptimal food were not in line with the results of previous studies which showed that vegetarians have a lower habit of wasting food [32,33] or a stronger attitude towards waste prevention [34]. Another study found that consumers who prefer vegetarian dishes have a greater willingness to consume ageing (suboptimal) products [35]. A possible explanation for the difference between the findings of this study and other studies is that vegetarians may be more health conscious and place more emphasis on food quality [36,37]. Therefore, they are more concerned about the quality of suboptimal food and prefer to throw it away.

The results on the association between quality perception and the acceptance of suboptimal food from this study show that consumers describe all three types of suboptimal food as “not attractive/tempting to eat/drink” and also “to be consumed as soon as possible”. This indicates that the majority of consumers perceive suboptimal foods as unattractive and also feel the need to consume them as quickly as possible to avoid wastage. Furthermore, the results of this study on quality perception and suboptimal food were consistent with the findings of de Hooge et al., 2017, which also showed an association between unattractiveness and suboptimal food. In contrast to the results of de Hooge et al., 2017, the urgency of consuming suboptimal foods did not show a significant association with most types of suboptimal foods [8].

This study was the first study conducted in Malaysia on consumers’ quality perception and acceptance of suboptimal foods that deviated visually, temporally, and peripherally. However, it is important to acknowledge the limitations of this study. First, there is the methodological limitation of an online survey, which is only completed by people who are computer-literate and have internet access. Consequently, the sociodemographic characteristics of the consumers in this study were not evenly distributed, which could impact the generalisability of the results. Specifically, there was a higher proportion (three-quarters) of female consumers in this study, and the majority were single, young adults with educational backgrounds ranging from matriculation/diploma/STPM/foundation to bachelor’s degrees. Another limitation is the geographical coverage, as this study focused exclusively on consumers living in Selangor or Kuala Lumpur. Therefore, the results of this study may not be representative of the entire population of Malaysia. However, our study provides important insights into suboptimal food among young Malaysian consumers who are the future of the nation.

## 5. Conclusions

In general, consumers in Selangor and Kuala Lumpur have a low acceptance of suboptimal foods that are visually, temporally, and peripherally deviant. The quality of suboptimal food was perceived by consumers as unattractive and that it should be consumed quickly. Furthermore, sociodemographic factors such as ethnicity, household income, and diet practice influenced the acceptance of suboptimal food and the likelihood of discarding suboptimal food.

The findings of this study provide important implications for decision-makers in developing strategies and interventions to effectively reduce food waste by increasing consumer acceptance of suboptimal food. Tailoring education and awareness programs to the specific sociodemographic characteristics of target consumers is crucial in achieving successful outcomes. Factors such as ethnicity, household income, and dietary patterns should be considered when designing and implementing initiatives to maximise the relevance and effectiveness of food waste reduction efforts. This study recommends that policymakers, food manufacturers, and retailers incorporate these findings into their initiatives to combat food waste. By taking into account the different sociodemographic profiles of consumers, they can tailor their messaging, marketing, and product offerings to different populations to ensure the success of food waste reduction programs. Despite the valuable insights provided by this study, it is important to acknowledge that, due to the use of a convenience sampling method, the results may have limited generalisability to the entire population in Malaysia. Decision-makers should be mindful of this limitation and consider further research with more diverse and representative samples to validate and extend these findings.

In future studies, it would be valuable for researchers to explore different types of suboptimal foods and their impact on consumer perceptions and acceptance, taking into account visual, temporal, and peripheral dimensions, to determine if they respond similarly to the findings of this study. For example, investigating consumers’ perceptions and acceptance of visually flawed suboptimal foods such as cucumbers or carrots would provide insight into whether consumers extend their aversion to visually flawed suboptimal fruits to visually flawed suboptimal vegetables. Similarly, examining suboptimal foods that are approaching their expiry date, such as bread or packaged juices, would shed light on how consumers perceive and accept suboptimal food products, other than suboptimal milk, that deviate in time. By exploring a broader range of suboptimal food types, researchers can gain a more comprehensive understanding of consumer perceptions and acceptance of suboptimal foods, contributing to the development of targeted strategies and interventions that address different suboptimal food categories and effectively promote consumer acceptance to reduce food waste.

## Figures and Tables

**Table 1:** Sociodemographic characteristics of the consumers, *n* = 414.

Sociodemographic Characteristics	*n* (%)
Gender	
Male	88 (21.3)
Female	326 (78.7)
Age	
18–29 years old	391 (94.4)
30–59 years old	23 (5.6)
Ethnicity	
Malay	108 (26.1)
Chinese	266 (64.3)
Indian	27 (6.5)
Others	13 (3.1)
State	
Selangor	299 (72.2)
Kuala Lumpur	115 (27.8)
Marital status	
Single	395 (95.4)
Married	17 (4.1)
Divorce	2 (0.5)
Monthly household income ^1^	
Less than RM 4849	284 (68.6)
RM 4850–RM 10959	87 (21.0)
More than RM 10959	43 (10.4)
Household size	
1–3 people	94 (22.7)
4–6 people	267 (64.5)
7–9 people	31 (7.5)
More than 9 people	3 (0.7)
Not specified	19 (4.6)
Highest education level	
No formal education	1 (0.2)
Primary school	1 (0.2)
Secondary school	30 (7.2)
Matriculation/diploma/STPM/foundation	201 (48.6)
Bachelor’s degree	157 (37.9)
Master’s or doctoral degree	24 (5.8)
Dietary patterns	
Vegan diet	10 (2.4)
Vegetarian	17 (4.1)
Flexitarian	74 (17.9)
Varied diet with meat and/or fish	309 (74.6)
Others	2 (0.5)
Not specified	2 (0.5)

^1^ Source: Household Income and Basic Amenities Survey Report 2019, Department of Statistics Malaysia. RM, Malaysian Ringgit; 1 USD = RM 4.44 as of 25 April 2023.

**Table 2:** Acceptance of three types of suboptimal food (A: optimal; B: suboptimal) at home and in the supermarket, the possibility of suboptimal food being discarded, and the promotion threshold to purchase suboptimal food.

Suboptimal Food	Setting						Possibility of Discarding Suboptimal Food ^1^ (*n* = 364) Mean ± SD	Promotion Threshold to Purchase Suboptimal Food ^2^ (*n* = 364) Mean ± SD
	Overall (*n* = 728) *n* (%)		Home (*n* = 364) *n* (%)		Supermarket (*n* = 364) *n* (%)			
	A	B	A	B	A	B		
Apple (visual)	656 (90.1)	72 (9.9)	299 (82.1)	65 (17.9)	357 (98.1)	7 (1.9)	4.56 ± 2.96	66.7 ± 25.1
Milk (temporal)	655 (90.0)	73 (10.0)	305 (83.8)	59 (16.2)	350 (96.2)	14 (3.8)	7.20 ± 3.21	77.3 ± 25.5
Biscuits (peripheral)	623 (85.6)	105 (14.4)	265 (72.8)	99 (27.2)	358 (98.4)	6 (1.6)	3.66 ± 3.27	61.0 ± 27.9
Average	644.7 (88.6)	83.3 (11.4)	289.7 (79.6)	74.3 (20.4)	355 (97.5)	9 (2.5)	5.14 ± 3.49	68.3 ± 27.0
*p*-value	0.008 *		*p* < 0.001 **		0.115		*p* < 0.001 **	*p* < 0.001 **

The *p*-value was obtained by a Chi-square or Kruskal–Wallis test to compare the acceptance, possibility to discard, and promotion threshold between three types of suboptimal food. * Significant at *p* < 0.05; ** significant at *p* < 0.001. ^1^ Scale from 0 to 10, where 0—would definitely be consumed; 5—equal chance of being consumed or discarded; and 10—would definitely be discarded. ^2^ Scale from 0 to 100, where 0—regular price or 0% discount; 50—half price or 50% discount; and 100—free or 100% discount.

**Table 3:** Awareness of food waste, estimation of food waste in the household, and perceived importance of reducing food waste in consumers.

	*N*	Mean ± SD	Median
Food waste awareness			
Estimated world’s food waste (%)	358	53.23 ± 21.05	52.5
Estimated consumer food waste (%)	359	41.49 ± 21.57	40.0
Estimation of food waste in the household			
Fresh fruits and vegetables (%)	362	21.02 ± 20.57	12.5
Milk and dairy (%)	362	16.00 ± 19.43	10.0
Bread and other bakery products (%)	362	16.46 ± 20.24	10.0
Meat and fish (%)	362	11.54 ± 17.27	5.0
Prepared dishes/meals (%)	362	26.42 ± 24.46	20.0
Average	362	18.29 ± 17.00	14.0
Relative importance of food waste compared to ^1^			
Reducing obesity	364	5.78 ± 1.25	-
Reducing pollution	364	5.50 ± 1.43	-
Stabilizing the economy	364	5.45 ± 1.48	-
Average	364	5.58 ± 1.18	-

^1^ Scale from 1 to 7, where 1 was “much less important” and 7 was “much more important”.

**Table 4:** Comparison between the acceptance of suboptimal food and the sociodemographic characteristics of consumers.

Sociodemographic	Suboptimal Food (B)					
	Overall (*n* = 2184) *n* (%)	*p*-Value	Home (*n* = 1092) *n* (%)	*p*-Value	Supermarket (*n* = 1092) *n* (%)	*p*-Value
Gender		0.454		0.232		0.351
Men	51 (10.5)		43 (17.7)		8 (3.3)	
Female	199 (11.7)		180 (21.2)		19 (2.2)	
Age		0.162		0.284		0.395
18–29 years old	241 (11.7)		214 (20.7)		27 (2.6)	
30–59 years old	9 (7.5)		9 (15.0)		0 (0.0)	
Ethnicity		*p* < 0.001 **		*p* < 0.001 **		0.906
Malay	42 (7.1)		35 (11.9)		7 (2.4)	
Non-Malay	208 (13.0)		188 (23.6)		20 (2.5)	
State		0.449		0.156		0.136
Selangor	185 (11.8)		169 (21.5)		16 (2.0)	
Kuala Lumpur	65 (10.6)		54 (17.6)		11 (3.6)	
Marital status		0.691		0.678		0.680
Single	239 (11.5)		212 (20.4)		27 (2.6)	
Married	9 (9.4)		9 (18.8)		0 (0.0)	
Divorce	2 (16.7)		2 (33.3)		0 (0.0)	
Monthly household income		0.124		0.021 *		0.355
Less than RM 4849	161 (10.7)		139 (18.5)		22 (2.9)	
RM 4850–RM 10959	63 (14.2)		60 (27.0)		3 (1.4)	
More than RM 10959	26 (11.1)		24 (20.5)		2 (1.7)	
Household size		0.570		0.965		0.128
1–5 people	191 (11.5)		174 (20.9)		17 (2.0)	
More than 5 people	51 (12.5)		43 (21.1)		8 (3.9)	
Highest education level						
Completion of a diploma equivalent or lower	152 (12.0)	0.335	139 (22.0)	0.139	13 (2.1)	0.295
Completion of a bachelor’s degree or higher	98 (10.7)		84 (18.3)		14 (3.1)	
Dietary patterns		0.639		0.548		0.110
Vegan/vegetarian	13 (9.0)		12 (16.7)		1 (1.4)	
Flexitarian	45 (11.7)		36 (18.8)		9 (4.7)	
Varied diet	190 (11.6)		173 (21.1)		17 (2.1)	

The *p*-value was obtained by the Chi-square test or Fisher’s Exact test. * Significant at *p* < 0.05; ** significant at *p* < 0.001.

**Table 5:** Comparison between sociodemographic characteristics and the possibility of suboptimal food being discarded and the promotion threshold for purchasing suboptimal food.

Sociodemographic	Suboptimal Food (B)			
	Possibility of Discarding Suboptimal Food ^1^ (*n* = 364) Mean	*p*-Value	Promotion Threshold to Purchase Suboptimal Food ^2^ (*n* = 364) (%)	*p*-Value
Gender		0.114		0.029 *
Men	4.81		63.5	
Female	5.23		69.7	
Age		0.827		0.091
18–29 years old	5.14		67.9	
30–59 years old	5.13		75.7	
Ethnicity		0.105		0.318
Malay	5.48		66.5	
Non-Malay	5.01		69.0	
State		0.752		0.879
Selangor	5.12		68.8	
Kuala Lumpur	5.20		67.2	
Marital Status		0.757		0.179
Single	5.16		68.0	
Married	4.79		72.9	
Divorce	4.33		86.7	
Monthly household income		0.066		0.789
Less than RM 4849	5.31		68.0	
RM 4850–RM 10959	4.86		68.3	
More than RM 10959	4.54		70.3	
Household size		0.466		0.194
1–5 people	5.04		67.8	
More than 5 people	5.32		70.4	
Highest education level				
Completion of a diploma equivalent or lower	5.11	0.843	68.8	0.994
Completion of a bachelor’s degree or higher	5.18		67.7	
Dietary patterns		*p* < 0.001 **		0.515
Vegan/vegetarian	6.38		72.8	
Flexitarian	5.85		67.9	
Varied diet	4.84		68.0	

The *p*-value was obtained by a Mann–Whitney U test or Kruskal–Wallis test. * Significant at *p* < 0.05; ** significant at *p* < 0.001. ^1^ Scale from 0 to 10, where 0—would definitely be consumed; 5—equal chance of being consumed or discarded; and 10—would definitely be discarded. ^2^ Scale from 0 to 100, where 0—regular price or 0% discount; 50—half price or 50% discount; and 100—free or 100% discount.

**Table 6:** Association between quality perception and the acceptance of suboptimal food (B).

Quality Perception	Suboptimal Food					
	Apple (B)		Milk (B)		Biscuits (B)	
	Exp(b) (95% CI)	R²	Exp(b) (95% CI)	R²	Exp(b) (95% CI)	R²
Good taste	1.596 (0.803–3.172)	0.363	0.976 (0.359–2.654)	0.332	0.769 (0.421–1.406)	0.424
Bad taste	0.901 (0.349–2.325)		0.643 (0.289–1.432)		1.023 (0.180–5.817)	
Same taste as the other product	1.264 (0.681–2.346)		0.947 (0.457–1.966)		3.906 (1.975–7.726) **	
Safe to eat/drink	1.012 (0.515–1.990)		1.234 (0.574–2.654)		1.719 (0.939–3.145)	
Not safe to eat/drink	0.397 (0.120–1.311)		0.627 (0.283–1.390)		0.150 (0.015–1.475)	
Not attractive/tempting to eat/drink	0.317 (0.170–0.590) **		0.371 (0.168–0.819) *		0.595 (0.357–0.991) *	
Suitable for adults	2.038 (1.032–4.024) *		1.453 (0.624–3.383)		0.675 (0.292–1.559)	
Suitable for children	2.144 (0.830–5.536)		1.541 (0.360–6.599)		1.391 (0.581–3.328)	
Suitable for serving to guests	0.817 (0.217–3.083)		1.548 (0.318–7.543)		2.385 (0.956–5.951)	
To be discarded	0.355 (0.073–1.726)		0.436 (0.194–0.981) *		0.782 (0.153–3.992)	
To be consumed as soon as possible	2.243 (1.153–4.361) *		3.345 (1.666–6.716) **		1.790 (1.068–3.000) *	
To be used in cooking	1.194 (0.599–2.380)		1.069 (0.463–2.472)		1.310 (0.654–2.624)	

The *p*-value was obtained by a binary logistics regression test. The hit rate (% of observations that are correctly predicted by the model) for apples (B), milk (B), and biscuits (B) was 91.5%, 92.0%, and 85.6%, respectively. * Significant at *p* < 0.05; ** significant at *p* < 0.001.

## Data Availability

The data presented in this study are available on request.

## Supplementary Materials

The following supporting information can be downloaded at: https://www.mdpi.com/article/10.3390/foods12152824/s1, File S1: Stimuli and scales for suboptimal food selection at home and in the supermarket.
