# Supplementary material for: Consumers’ Quality Perception and Acceptance of Suboptimal Food: An Online Survey in Selangor and Kuala Lumpur, Malaysia
Source: Foods. 2023 Jul 25;12(15):2824. doi: 10.3390/foods12152824 (PMC10416839; doi:10.3390/foods12152824)
Supplement: Supplementary file 1 [file foods-12-02824-s001.zip › foods-2461120-supplementary.pdf]

Supplementary materials

Stimuli and scales for suboptimal food selection at home and in the supermarket

Situation: At home

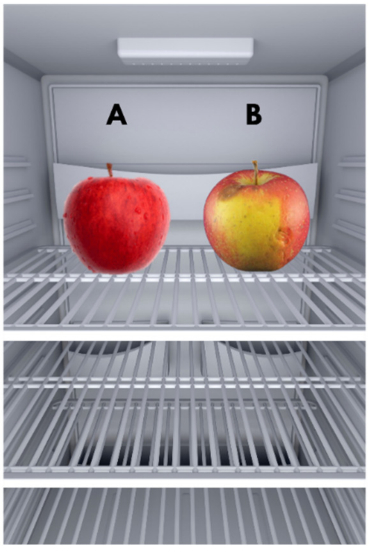

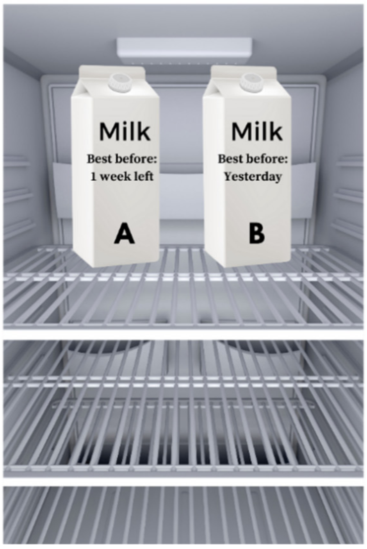

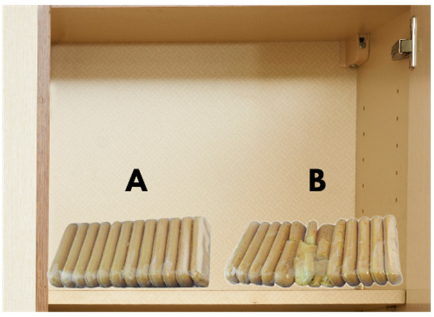

1. Imagine that you're in your home, ready to select [an apple/ milk/ biscuits]. Which one would you choose?

( ) A

( ) B

( ) None of these

2. In your household, how probable is it that [apple B/ milk B/ biscuits B] would be discarded in the garbage?

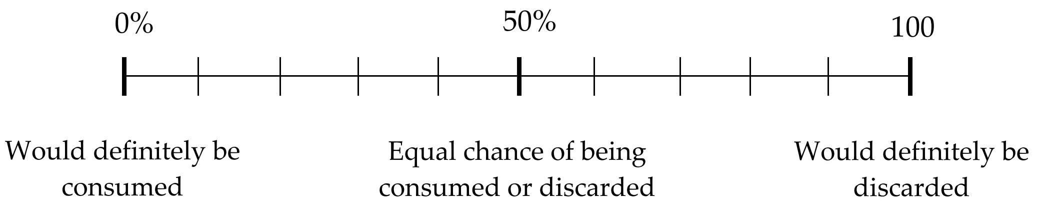

3. Please describe [apple B/ milk B/ biscuits B]. Check all the alternatives that apply to this particular product.

( ) Good taste

( ) Bad taste

( ) Same taste as the other product

( ) Safe to eat/ drink

( ) Unsafe to eat/ drink

( ) Not attractive/ tempting to eat/ drink

( ) Suitable for adults

( ) Suitable for children

( ) Suitable for serving to guests

( ) To be discarded

( ) To be consumed as soon as possible

( ) To be used in cooking

Situation: In the supermarket

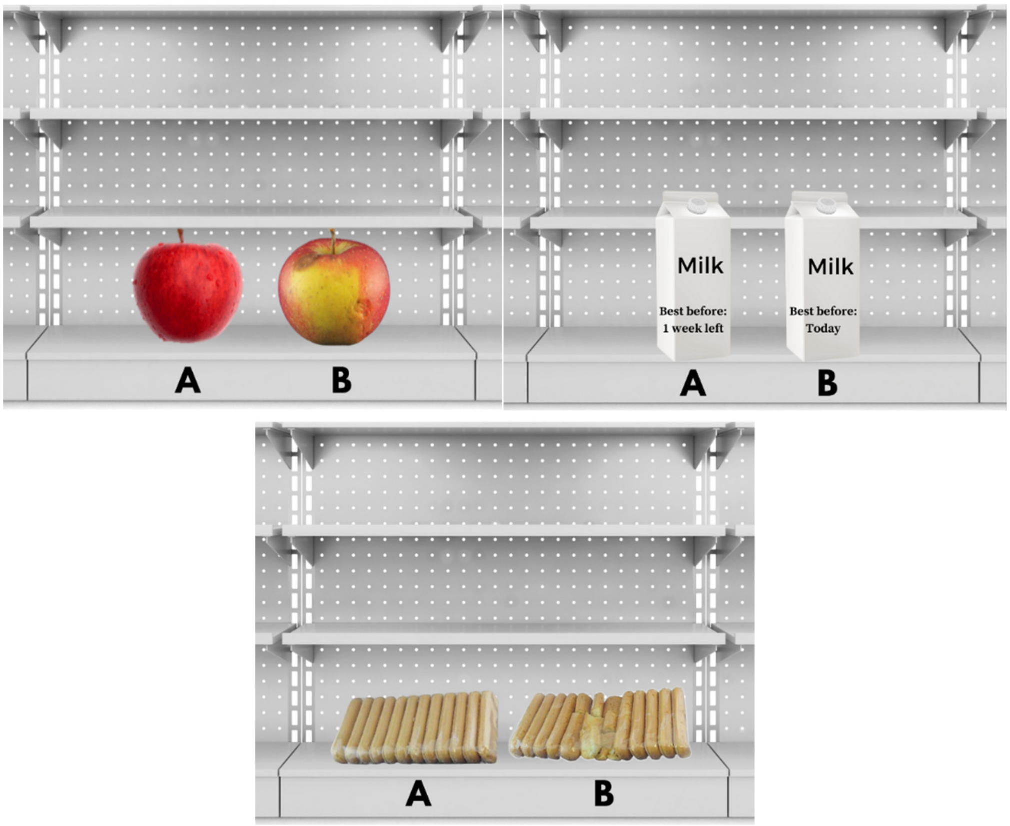

1. Imagine that you're in a supermarket, ready to select [an apple/ milk/ biscuits]. Given an identical price, which one would you choose?

( ) A

( ) B

( ) None of these

2. For which discount would you purchase [apple B/ milk B/ biscuits B]? Indicate your highest acceptable price.

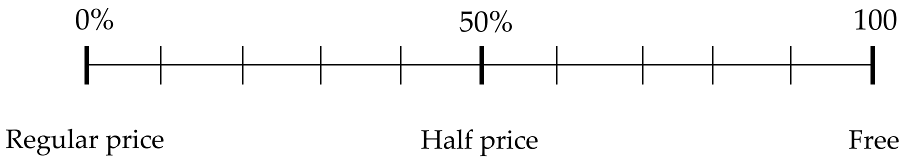

3. Please describe [apple B/ milk B/ biscuits B]. Check all the alternatives that apply to this particular product.

( ) Good taste

( ) Bad taste

( ) Same taste as the other product

( ) Safe to eat/ drink

( ) Unsafe to eat/ drink

( ) Not attractive/ tempting to eat/ drink

( ) Suitable for adults

( ) Suitable for children

( ) Suitable for serving to guests

( ) To be discarded

( ) To be consumed as soon as possible

( ) To be used in cooking
